# Unique sperm haplotypes are associated with phenotypically different sperm subpopulations in *Astyanax* fish

**DOI:** 10.1186/s12915-018-0538-z

**Published:** 2018-07-05

**Authors:** Richard Borowsky, Alissa Luk, Xinjian He, Rebecca S. Kim

**Affiliations:** Department of Biology, New York University, New York, USA

**Keywords:** Sperm competition, Sib sperm competition, *Astyanax mexicanus*, Cave fish, Sperm phenotypes

## Abstract

**Background:**

The phenotypes of sperm are generally believed to be under the control of the diploid genotype of the male producing them rather than their own haploid genotypes, because developing spermatids share cytoplasm through intercellular bridges. This sharing is believed to homogenize their content of gene products. However, not all developing spermatids have identical gene products and estimates are that alleles at numerous gene loci are unequally expressed in sperm. This provides scope for the hypothesis that sperm phenotypes might be influenced by their unique haplotypes. Here we test a key prediction of this hypothesis.

**Results:**

The haploid hypothesis predicts that phenotypically different sperm subpopulations should be genetically distinct. We tested this by genotyping different sperm subpopulations that were generated by exposing sperm to a chemical dye challenge (Hoechst 33342). Dye treatment caused the cells to swell and tend to clump together. The three subpopulations of sperm we distinguished in flow cytometry corresponded to single cells, and clumps of two or three. Cell clumping in the presence of the dye may reflect variation in cell adhesivity. We found that allelic contents differed among the three populations. Importantly, the subpopulations with clumped sperm cells were significantly enriched in allelic combinations that had previously been observed to have significantly lower transmission success.

**Conclusions:**

We show that at least one sperm phenotype is correlated with its haploid genotype. This supports a broader hypothesis that the haploid genotypes of sperm cells may influence their fitness, with potentially significant implications for the transmission of deleterious alleles or combinations of alleles to their offspring.

**Electronic supplementary material:**

The online version of this article (10.1186/s12915-018-0538-z) contains supplementary material, which is available to authorized users.

## Background

There is a rich literature on sperm competition based on the evolutionary insight that the competition between males to fertilize a female does not necessarily end with insemination [1]. Thus, in many species, there is opportunity for sperm from different males to compete for successful fertilization of the ova [2, 3]. This inter-individual sperm competition helps drive the evolution of more competitive sperm within a species through selection at the level of diploid genotypes. However, the competing entities, individual sperm cells, are haploid. Thus, we ask to what extent is an individual sperm’s likelihood of fertilization success dependent on its haploid genotype? This question is not only relevant to competition between sperm from different males, but also to competition between sperm from the same male [4]. In fact, the latter situation, sib sperm competition, is far more common than competition between sperm from different males. Here, we test the hypothesis that the haploid genotypes of individual sperm influence their phenotypes by testing one of its key predictions, that sperm of different phenotypes differ in genotype.

At first glance the haploid control hypothesis seems improbable. Developing spermatids share cytoplasm through intercellular bridges which potentially homogenizes all their contents [5–7]. Thus, it has been argued that while “genetically haploid”, sperm cells are “phenotypically diploid” [5]. Nevertheless, the homogenization is not universal [8, 9] and hundreds of genes are estimated to have unequally expressed alleles in sperm from the same male [10]. Thus, genetic variability among the sperms’ haploid genotypes could account for significant phenotypic variability among sperm from an individual male. This view is strongly supported by a recent study comparing subpopulations of sperm from single zebrafish males [11]. The study had two key findings: first, that ova fertilized by longer-lived sperm had greater survival rates and developed into more robust adults than those fertilized by shorter-lived sperm. In addition, genomic analyses showed that faster swimming sperm differed from slower swimming sperm in allelic content at numerous sites across the genome. Although the relevant genes were not identified, these results clearly established that the haploid genomes of sperm can influence adaptively significant phenotypes of sperm. Here we explore this phenomenon and identify two candidate genes which were involved in adaptation to cave life and show that allelic content at these two loci are significantly associated with sperm phenotype.

Previously [12], we presented evidence for epistatic interactions among unlinked loci in a mating between F_1_ hybrids derived from a cross between a cave morph (C) and a surface morph (S) of *Astyanax mexicanus*, the Mexican Tetra. These interactions were manifested as departures from Mendelian expectations of independent assortment in the transmission of alleles from the F_1_ to the F_2_. For loci A and B, for example, significant excesses of homospecific combinations (A_C_B_C_ or A_S_B_S_) over heterospecific combinations (A_C_B_S_ or A_S_B_C_) were observed. (Hereafter, the proportions of homospecific and heterospecific combinations are referred to as HOM and HET, respectively.) The most strongly interacting loci we studied were *oca2* and *mc1r* [12]. In this paper, we take a closer look at the relationships between allelic combinations at these two loci and sperm phenotype.

## Results

### The phenotype

**Fig. 1:**
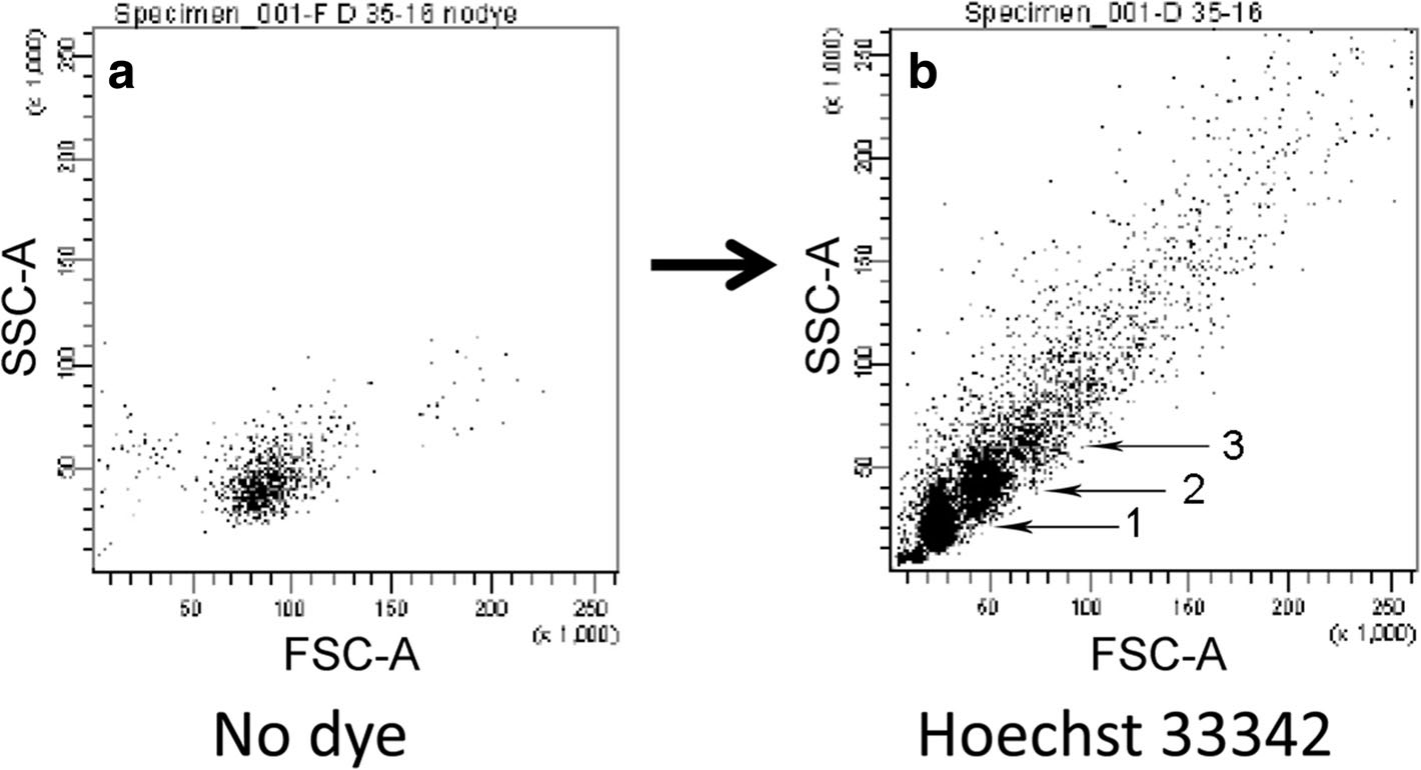
Hoechst 33342 dye treatment typically reveals three subpopulations of hybrid male sperm in flow cytometry. **a** Sperm from a hybrid male form one population in 0.4× Hanks balanced saline. **b** Sperm from the same male treated with Hoechst 33342 dye (40 μM) form three subpopulations indicated by arrows

The haploid control hypothesis predicts that sperm of different phenotypes should differ in their allelic contents. We have found that a simple and reliable phenotype for *A. mexicanus* sperm is their response to a chemical challenge. Untreated sperm of hybrids form one distinct population in flow cytometry (Fig. 1a) while sperm treated with Hoechst 33342 (40 μM in Hank’s buffer) typically display three (or more) distinct subpopulations (Fig. 1b). The three subpopulations are distinguished in visible light by Forward Scatter (FSC-A) and Side Scatter (SSC-A). Hoechst fluorescence reveals that subpopulation 1 (closest to the origin) consists of single cells, while subpopulations 2 and 3 correspond to clusters of cells, specifically, doubletons and triplets (Additional file 1).

We examined dye-treated and untreated sperm from single males with the light microscope (phase contrast) and found significant differences between treatments (Methods). Untreated sperm were almost uniformly complete with a translucent head, a visible midpiece, and a flagellum. The head was spherical and measured 3.78 ± 0.11 μm SEM (*n* = 32). Dye-treated sperm exhibit more structural diversity, having complete sperm but also those missing midpiece and flagellum. The head sizes of the complete dye-treated sperm (3.68 ± 0.04 μm, SEM, *n* = 23) and the ones with missing parts (3.63 ± 0.04 μm, SEM, *n* = 28) are nearly identical. The incomplete sperm vary in translucence from bright, as exhibited by untreated sperm, to dim translucence that is just noticeable. In addition to these, there is a class of dark, clearly necrotic, cells that are recognizable by their complete lack of translucence and their granular structure. The dark cells are significantly larger (4.85 ± 0.06 μm, SEM, *n* = 61) than the complete or translucent cells (*t* = 9.7, df = 91; *t* = 12.2, df = 82; *t* = 13.7, df = 87; all *p* values < 10E−6).

We compared treated and untreated sperm in two experiments. Only small proportions of the total cells were necrotic in the untreated samples (0.024 and 0.0032, experiments 1 and 2, respectively), but the proportions of necrotic cells were significantly and dramatically higher in the dye-treated samples (0.395 and 0.145, respectively) (experiment 1: *χ*^2^ = 108.4, df = 1, *p* << 0.0001; experiment 2: *χ*^2^ = 183.2, df = 1, *p* << 0.0001).

The addition of dye also led to a dramatic increase in the number of cell clustering. The clusters consisted of two cells (the most common configuration) to higher levels up to six cells. In experiment 1, the percentage of cells in clusters in the untreated samples was 2/206 = 1.0% and in the dye-treated cells was 39/185 = 21.1% (*χ*^2^ = 39.9, df = 1, *p* << 0.0001). In experiment 2, the comparable figures were 21/620 = 3.4% and 625/1333 = 46.9% (*χ*^2^ = 359.8, df = 1, *p* << 0.0001).

Focusing on doubletons in dye-treated samples, in experiment 1, the doubletons make up about 11% of doubletons plus singles. In experiment 2, the percentage is 20.2%. In flow cytometry (FACS Diva), the average percentage of the gated subpopulation 2 was 11.3 ± 6.7% of the total number of events. The agreement between FACS and microscopy is in accord with the expectation from the Hoechst fluorescence data (Additional file 1) that the doubletons counted under the microscope and the subpopulation 2 events detected in flow cytometry are the same populations.

Clusters included both translucent and dark cells, although they were significantly enriched in dark cells. Focusing on the dye-treated samples, in experiment 1, the representation of dark cells in singletons was 50% (*n* = 146) but in clusters was 72% (*n* = 36, *χ*^2^ = 4.89, df = 1, *p* = 0.027; Additional file 2). In experiment 2, the representations were 27% in singletons (*n* = 708) and 37% in clusters (*n* = 641, *χ*^2^ = 12.87, df = 1, *p* = 3.3E−4; Additional file 2). This enrichment suggests that clustering is driven disproportionately by the dark, necrotic cells.

In summary, the responses of the sperm cells to the dye were at least twofold; first, many of the sperm swell significantly and appear necrotic while others are more resistant. Second, there is an increased tendency for cells to clump. The dye treatment is clearly a challenge to *Astyanax* sperm, but not every cell succumbs. We propose that the variability in response reflects the inherent general fragility of the sperm cell and that subpopulations 2 and 3, as detected by FACS, are enriched in the more fragile cells. The question is does this phenotype correlate with the haploid genotypes of the cells?

### Genotyping

We used flow cytometry and FACS to collect sperm from the three subpopulations (as in Fig. 1b) and tested them for significant enrichment with HOM or HET combinations of alleles at the *oca2* and *mc1r* loci. We chose these loci to investigate because of their strong epistatic interactions in our original study [12]. Additionally, the protein products of both loci are membrane bound and both are involved in reduction of pigmentation during cave adaptation in this species [13, 14]. Thus, their shared venue and functional relationship strengthen the possibility that they may be true epistatic interactors.

For sperm sources, we used 10 different sibling offspring of a surface morph female and a Pachón cave morph male. Two of the 10 males each provided two samples. We attempted to deposit single sperm cells from each of the three subpopulations into wells of 96-well PCR plates using FACS. We genotyped the samples in two stages: first, the contents of each well received a whole genome primer extension preamplification (PEP [15, 16]) to obtain sufficient DNA for PCR. The PEP products were analyzed by qPCR using allele specific primers and probes (Additional file 3). We used the end-point genotyping function of LightCycler480 software to estimate the relative allelic proportions at both loci in each well. We ran multiple replicate 96-well plates: 10 for subpopulation 1, 12 for subpopulation 2, and 8 for subpopulation 3. The data were pooled within subpopulations for analysis (Methods). The results indicated that while some wells received single cells, the majority had received more than one sperm. Because wells having received many sperm would be uninformative, we screened the results to limit inclusion for analysis to those wells having received one or a small number of sperm. The screen was based on a trigonometric conversion of the qPCR endpoint data with stringency defined by an input angle (Methods). We focused on wells in which the genotypes for both loci appeared to be haploid or those in which the well contents strongly leaned towards one allele or the other. For inclusion of a well in the analysis, both loci had to meet the criterion. If both loci were typed as cave or as surface, the well was designated HOM. If not, it was designated HET. The expected proportion of HET under the null hypothesis is 0.5. Statistical significance was determined using the binomial test. Haplotype frequencies in the three subpopulations were also directly compared in a *χ*^2^ contingency analysis.

When the stringency for inclusion was tightest (15°), only about 2% of the data passed the screen. Stringencies were relaxed in six steps to include greater numbers of wells, to a maximum inclusion of about 13.5% of the total dataset (30°). For subpopulation 1, at all levels of stringency, HET:HOM did not deviate significantly from the expected ratio of 1:1 (Table 1). For subpopulation 2, HET was significantly greater than HOM at the three most inclusive stringencies (*p* ranging from 0.05 down to 2.49E−5). For subpopulation 3, HET was significantly greater than HOM at six of the seven stringency levels (*p* ranging from 0.036 down to 0.0029). Combining the data from subpopulations 2 and 3, HET significantly exceeded HOM at four of the stringency levels (*p* ranging from 0.015 down to 2.00E−6). The ratio of HET:HOM in the combined data set at the most inclusive stringency was 0.647:0.343.

**Table 1:** Subpopulations of sperm differ in allelic content

	Filter	% of Dataset				Two-tailed
	Angle (degrees)	Retained	HET	HOM	P(HET)	Binomial *p* =
SubPop 1	15	2.8	14	13	0.519	1
	17.5	3.1	17	13	0.567	0.585
	20	3.9	20	17	0.541	0.743
	22.5	4.8	24	22	0.522	0.883
	25	6.6	31	32	0.492	1
	27.5	9	37	49	0.430	0.235
	30	12.6	54	67	0.446	0.275
SubPop 2	15	1.6	10	9	0.526	1
	17.5	2	11	12	0.478	1
	20	2.7	17	14	0.548	0.72
	22.5	4.2	29	19	0.604	0.193
	25	5.8	42	25	0.627	0.05
	27.5	8.9	67	36	0.650	2.92E−03
	30	13.5	104	51	0.671	2.49E−05
SubPop 3	15	1.3	9	1	0.900	0.0215
	17.5	2.3	12	6	0.667	0.238
	20	3.7	20	8	0.714	0.035
	22.5	4.3	23	10	0.697	0.0351
	25	6.3	31	17	0.646	0.059
	27.5	9.4	49	23	0.681	0.00294
	30	14.5	68	43	0.613	0.022
SubPops 2 and 3	15	1.5	19	10	0.655	0.136
	17.5	2.1	23	18	0.561	0.533
	20	3.1	37	22	0.627	0.067
	22.5	4.2	52	29	0.642	0.015
	25	6	73	42	0.635	0.00511
	27.5	9.1	116	59	0.663	1.97E−05
	30	13.9	172	94	0.647	2.00E−06

The three subpopulations are defined in the text. The angle sets the stringency of the filter to screen out intermediate wells. The stringency’s affect is reflected in the percentage of wells in the dataset that pass the filter and are retained for downstream analysis. Numbers of wells determined by genotyping to have HET or HOM combinations and the proportion of HET are listed in columns 4 to 6. The last column lists the two tailed binomial probability of the null hypothesis: P(HET) = P(HOM). In subpopulations 2 and 3, but not in subpopulation 1, there are significant departures from the null, with P(HET) > P(HOM)

Subpopulations 2 and 3, which are enriched with sperm cells most affected by the dye treatment, are also enriched in heterospecific combinations of alleles at *mc1r* and *oca2*. Thus, there is a connection between the haploid genotypes of sperm cells and their phenotypes.

### The two HET combinations differ in their properties

In order to determine whether only one or both HET combination are enriched in subpopulations 2 and 3, we enumerated all four haplotypes using the data from the most inclusive filter (i.e., 30°). This admitted 13.4% of all the wells (*n* = 387) for analysis. The four haplotypes are the combinations of alleles (**C**ave vs **S**urface) at the two loci (***M****c1r* and ***O****ca2*). Table 2a is a 3 × 4 contingency table which shows that the distribution of haplotypes among subpopulations is not random (*χ*^2^ = 105.3, df = 6, *p* < 1E−6). Some haplotypes are overrepresented in a particular subpopulation and others are underrepresented. To identify individual cells as significant contributors, we calculated standardized residuals (*z*) for each cell as (observed − expected)/(expected^.5). *z* is distributed roughly as a normal deviate with a mean of zero and a standard deviation of 1.0. Thus, we considered any cell with an absolute *z* value greater than 2.0 as significant. For example, the HET combination M_S_ O_C_ has an expected frequency of 62.5 in subpopulation 2, but its observed frequency was 99 (*z* = 4.6, *p* < 1E−4). Thus, this haplotype is significantly overrepresented in that population. In contrast, the other HET combination, M_C_ O_S_, was underrepresented in the same subpopulation (*z* = − 4.4, *p* < 1E−4). This shows that not all HET combinations are suboptimal (i.e., overrepresented in the higher subpopulations).

**Table 2:** The distribution of haplotypes among the subpopulations of sperm

2a All three subpopulations (*χ*^2^ = 105.3, df = 6, *p* < 1E−6)				
Observed	M_C_ O_S_	M_S_ O_C_	M_C_ O_C_	M_S_ M_O_
Pop1	44	*10*	*19*	48
Pop2	5	*99*	*18*	33
Pop3	21	47	19	24
Expected	M_C_ O_S_	M_S_ O_C_	M_C_ O_C_	M_S_ M_O_
Pop1	*21.9*	*48.8*	17.5	*32.8*
Pop2	*28.0*	*62.5*	22.4	42.1
Pop3	20.1	44.7	16.1	30.1
Standardized residuals (*z*)				
	M_C_ O_S_	M_S_ O_C_	M_C_ O_C_	M_S_ M_O_
Pop1	4.7	−5.6	0.4	2.6
Pop2	−4.4	4.6	−0.9	−1.4
Pop3	0.2	0.3	0.7	−1.1
2b Sub populations 2 and 3 merged (*χ*^2^ = 87.7, df = 3, *p* < 1E−6)				
Observed	M_C_ O_S_	M_S_ O_C_	M_C_ O_C_	M_S_ M_O_
Pop1	*44*	*10*	19	*48*
Pops2&3	*26*	*146*	37	57
Expected	M_C_ O_S_	M_S_ O_C_	M_C_ O_C_	M_S_ M_O_
Pop1	*21.9*	*48.8*	17.5	*32.8*
Pops2&3	*48.1*	*107.2*	38.5	72.2
Standardized residuals (z)				
	M_C_ O_S_	M_S_ O_C_	M_C_ O_C_	M_S_ M_O_
Pop1	4.7	−5.6	0.4	2.6
Pops2&3	−3.2	3.7	−0.2	−1.8

The distribution of the four haplotypes among the subpopulations of sperm is not random. The haplotypes are *Mc1r* Cave/*Oca2* Surface (M_C_ O_S_), *Mc1r* Surface/*Oca2* Cave (M_S_ O_C_), *Mc1r* Cave/*Oca2* Cave (M_C_ O_C_), and *Mc1r* Surface/*Oca2* Surface M_S_ M_O._ Entries in italics are considered significant with *z* > 2

Table 2b shows the same data as Table 2a but simplifies the analysis by combining subpopulations 2 and 3. As in Table 2a, the distribution of haplotypes among subpopulations is not random (*χ*^2^ = 87.7, df = 3, *p* < 1E−6). The residual analysis clearly shows M_S_ O_C_ to be overrepresented in the combination of subpopulations 2 and 3 (observed = 146, expected = 107.2, *z* = 3.70) and underrepresented in subpopulation 1 (observed = 10, expected = 48.8, *z* = − 5.6). The opposite is the case for M_C_ O_S_, which is underrepresented in the combination of subpopulations 2 and 3 (observed = 26, expected = 48.1, *z* = − 3.2 and overrepresented in subpopulation 1 (observed = 44, expected = 21.9, *z* = 4.7).

In summary, our original expectation, that the representation of HET combinations in the different subpopulations is not random, is strongly supported by the data. This result is largely driven by the behavior of haplotype M_S_ O_C_. On the other hand, we did not anticipate that the different HET combinations could be either over- or underrepresented in populations 2 and 3. More research will be required to uncover a general pattern.

### Simulation of the experiment

We simulated the experiment to test whether the analysis could effectively distinguish HET from HOM wells, when they had received more than one sperm (details in Methods). We ran simulations with sets of 5 or 10 haplotypes, modeling the situation with experimental wells that had received 5 or 10 sperm.

Haplotypes were determined with unbiased input (null model) and at two levels of biased input. For the unbiased simulations, all four haplotypes had probabilities set at 0.25. For the biased simulations, the HET combinations both had probabilities set at 0.3 with both HOM combinations set at 0.2 (stronger bias), or at 0.275 and 0.225, respectively (weaker bias).

For each simulation, 1000 sets of haplotypes were generated. Each set is analogous to a single well in the actual experiments. For analysis, each “well” in the simulation was characterized by the number of cave alleles for *mc1r* and the number of cave alleles for *oca2*. That is, although the wells were originally based on 5 or 10 haplotypes, the linkage information was discarded and only the allelic proportions were retained for downstream analysis. This matches the situation in the actual experiments. Thus, the question was whether the analysis of allelic frequencies could recover useful information about the original haplotypic contents.

For the analyses, the 1000 replicates were screened to remove intermediates, as was done in analysis of the experimental data. For screening in the simulations, we used two filters: the more stringent filter discarded any well with a cave allelic frequency greater than 0.2 or less than 0.8, at either locus. In a less stringent filter, these values were at 0.3 and 0.7, respectively. The 10 cell simulations were screened at both levels but the 5 cell simulations could only be screened at the more stringent level. This procedure models the trigonometric screen for the analysis of the real experiments.

The percentage of wells that passed the filter in the simulations ranged from 1.0 to 16.5 (Table 3) and was almost identical to the range of percentages of wells that passed the screens in the experiments, 1.3 to 14.5 (Table 1). This suggests that the values chosen for the simulations were plausible. The wells that passed the filters were then designated HOM if the proportions of cave alleles at the two loci were both high or were both low. They were designated HET if one was high and the other was low.

**Table 3:** Computer simulation of the experimental analysis confirms its efficacy to detect biased haplotypic input

Model	Filter	% of dataset	HET:HOM	HET:HOM	Two-tailed
	Level	Retained	Input as P(HET)	Output	Binomial *p* =
5 cells	0.2–0.8	14.5	0.3	96:49	5.88E−05
		14.3	0.275	90:53	1.24E−03
		16.5	0.25	82:83	ns
10 cells	0.2–0.8	1.2	0.3	11:1	6.35E−03
		1.8	0.275	13:5	9.63E−02
		1.0	0.25	4:6	ns
10 cells	0.3–0.7	12.6	0.3	92:34	2.36E−07
		14.6	0.275	97:49	8.77E−05
		11.4	0.25	56:58	ns

The results of the simulation for models with 5 and 10 “cells” per “well.” Output bias of HET > HOM was tested using the binomial test. In each of the three simulations, when input was unbiased, no significant bias was exhibited in the output. In contrast, when input was biased (HET > HOM), significant bias of HET > HOM was detected in five of the six tests (*p* < 0.01)

The results of the simulations (Table 3) clearly show that the biased haplotypic inputs in both the 5- and 10-cell models allowed recovery of significantly biased allelic frequency output. It also showed that when the input was unbiased, no bias appeared in the output. Thus, the analysis is not creating spurious significance. The Excel files used for the simulations are interactive and are available as Additional files 4 and 5.

## Discussion

Our findings revealed that sperm from the same ejaculate can have different phenotype classes which contain different specific allelic combinations. In this case, the phenotype in dye-challenged samples was propensity to cluster in clumps of two or three (or more) cells. These clumps were enriched with the dark, necrotic cells, and our working hypothesis is that these cells are sticky.

Sperm clustering has been reported before [4, 17, 18] as a mechanism for active sib sperm to cooperate with one another and gain an advantage in fertilization. In our examination of untreated samples, we saw numerous intact sperm cells, but none of them clustered as in other studies [17]. The clumping phenomenon reported here is different because the cells that clump are damaged and incapable of fertilizing ova. In essence, the phenotype reported here would lead to exclusion from the fertilizing pool.

Earlier work showed that HOM combinations of *Oca2* and *Mc1r* alleles were transmitted preferentially from an F_1_ cave × surface hybrid [12]. The present study shows that subpopulations 2 and 3 are enriched in HET combinations, and their sperm cells may be more fragile than average. This suggests that such cells, under more natural conditions, might be less likely to be in the fertilizing pool than those from subpopulation 1. The cave and surface genomes represent different coadapted gene pools, so it might be expected that HOM combinations of alleles would be fitter than HET combinations, although, as the results show, not invariably.

As a caveat, we note that we chose *oca2* and *mc1r* to test for interaction because the differences between cave and surface populations had already been documented. But, while they are known to have related functions, we have no proof that their functions directly influence the interactions documented above; the relevant variation might be at other loci closely linked to the genes. This warrants further investigation.

The interactions between genes in haploid sperm need not be strong to have major effects on sperm fitness. Very small phenotypic differences in traits affecting sperm fitness are magnified in effect because of the huge numbers of competing individuals and the infinitesimal probabilities of individual success [19]. We suggest that sib sperm competition is potentially an important driver of evolutionary change because (1) it can magnify the selective effect of small phenotypic differences and (2) it operates on the haploid level and thus could efficiently screen deleterious recessives.

For a sib sperm competition based screen to drive an evolutionary response of increased zygotic fitness, there would have to be a correlation between an allele’s effect on both sperm and zygote fitness. There is some evidence for this in the literature. Immler et al. [20] separated salmon sib sperm based on their swimming longevity and showed that offspring derived from sperm with greater longevity had faster developmental rates than those derived from sperm with lesser longevity. These observations were extended in their study of zebrafish, in which they showed that zygotes fertilized by longer-lived sperm not only developed faster but also matured into more robust adults [11]. In competition between sperm of different male dung flies, sperm that were more successful fertilizers gave rise to faster developing offspring [21]. A correlation between copulation frequency and offspring fitness observed in adders suggested that “good genes” increased the probability of both fertilization success for sperm carrying them and zygote fitness [22]. The correlation between sperm and zygote fitness is widely accepted in the plant literature [23]; in angiosperms, one of the most important factors in competition among sperm cells is the rate at which the pollen tube grows. Mulcahy hypothesized that if the specific haploid genome of the gametophyte caused robust pollen tube growth it would likely contribute to robust growth in the resulting zygote [24, 25].

Sperm are remarkable and unique in their high proportions of malformed and malfunctioning cells. Morphologically abnormal sperm have been documented in humans and a wide variety of other animals [26–33]. It is generally believed that sperm cells are particularly difficult to manufacture correctly and that the large numbers of abnormal sperm cells represent “production errors” that eluded quality control [34]. In this view, the malformed sperm would represent wasted resources and be seen as lowering fitness.

Viewing this phenomenon in the light of evolution, however, suggests an alternative hypothesis. Perhaps the presence of malformed and other sub-optimal sperm is actually an important adaptation which, rather than decreasing fitness, raises fitness. Sib sperm competition might act as a screen against the transmission of deleterious alleles or allelic combinations (“bad genes”). If bad genes in the haploid genome of a sperm cell make it phenotypically inferior, those bad genes would be screened out. This would increase the proportion of good genes that would be in the fertilizing pool. Sib sperm competition could efficiently drive zygotic evolution, if advantageous alleles in sperm are also advantageous in the zygote [11, 22].

We consider how such a screen might evolve. Let us posit two alternatives: The basic sperm cell, manufactured under control of the diploid genotype of the male is robust in structure and function (“basic-robust”), as opposed to an alternative, that the basic model is inferior and just on the edge of non-functionality (“basic-borderline”). The basic-robust sperm would be able to be in the fertilizing pool no matter what genes were loaded into them. In contrast, the basic-borderline sperm would be easily pushed over the line to non-functionality by bad genes. That is, the screen against bad genes would not be effective if the sperm were basic-robust but could be quite effective if the sperm were basic-borderline. This would raise the overall quality of the genes transmitted and would bestow a fitness advantage on other genes, acting in the diploid phase, that would make for borderline sperm. This hypothesis is in direct contrast to the view that “from the perspective of the diploid genome of the male parent, all sperm are equally valuable” [35].

There may be evidence for sib sperm screening in an unwitting experiment: individual humans conceived by assisted reproductive techniques (ART), including in vitro fertilization and intracytoplasmic sperm injection (ICSI), are more prone to developmental abnormalities, up to 40% greater risk, than those conceived naturally [36–38]. While several possible explanations for this are debated in the literature, all forms of ART share the quality of greatly reducing or eliminating sib sperm competition. Thus, one possibility is that these poorer outcomes simply reflect the bypassing of sib sperm competition and its screen against deleterious genetics. If so, outcomes of ICSI, and ART generally, might be improved through employment of artificial screens mimicking the milieu in which sib sperm normally compete [39].

Finally, we note that while the evolutionary reproductive strategies of males and females differ, both sexes profit when sperm with the best genes fertilize the female’s ova. Thus, females, as well as males, should benefit from setting a high bar to the transmission of bad alleles [20, 40]. Birkhead et al. asked, “why do females make it so difficult for males to fertilize their eggs” [40]. Among other possibilities, they suggested that the rigors of the female reproductive tracts for sperm in both birds and mammals serve to increase the degree of competition among sperm, and bias fertilization success towards sperm of higher quality. We agree with this proposal. Their argument was framed in terms of traditional sperm competition theory (interejaculate competition [1]) but also applies to the more straightforward and far more common case of sib sperm competition (intraejaculate competition).

## Conclusions

Here we report that different phenotypic subpopulations of sperm from the same male differ in their allelic content. This demonstrates that the phenotypes of sperm cells are, at least partially, determined by their haploid genotypes. This observation runs counter to the prevailing dogma that sperm phenotypes are determined solely by the diploid genotypes of the males producing them [5]. We found that subpopulations of sperm cells that are enriched in fragile cells were also enriched in haplotypic combinations of alleles believed, based on previous work, to be suboptimal. If fragile sperm cells are less likely to be in the fertilizing pool than more robust cells, intra-ejaculate sib sperm competition could act as a potent screen against suboptimal alleles or allelic combinations. Because selection against recessives is far more efficient in haploid than in diploid stages, sib sperm competition could be a powerful engine of evolutionary change. In addition, our observations have implications for a central paradox of sperm biology, which is that there is typically a large proportion of non-functional sperm among the very cells essential for reproduction. The prevailing belief is that the high proportion of non-functional sperm cells reflects inadequate quality control in their manufacture and is maladaptive [34]. In contrast, we suggest that the high proportion of non-functional cells increases fitness by making the sib sperm competition screen more efficient in eliminating suboptimal haplotypes before they combine with the egg, leaving fertilization to genetically more robust sperm. Finally, our work has implications for assisted reproductive techniques (ART) in humans and domestic animals, because the various ART techniques all reduce or eliminate sib sperm competition and its potential to screen against deleterious genes. This may account for the observed increased risk of developmental disorders in children conceived though ART compared with those conceived naturally [36–38].

## Methods

### Sperm collection, dye treatment, and FACS methods

The temperature in the male’s tank, normally 21 °C, was increased over the course of 2 h to 26 °C to stimulate new sperm production [41]. They were held at 26 °C overnight and then gently squeezed the next day to collect sperm in Hank’s buffer [42]. Without the warm water treatment, some sperm can typically be obtained from males, but the treatment stimulates sperm production and results in copious amounts. Thus in all replicates of the experiment, we were working with sperm samples that were largely newly synthesized, and age equivalent. The suspension in buffer was diluted with 1.5 parts system water to a concentration of 0.4× Hank’s, and then brought up to a concentration of 40 μM Hoechst 33342 by addition of concentrated stock. Sperm were analyzed by FACS between 1 and 2.5 h after dye treatment. FACS was performed on a BD FACS Diva plotting events based on FSC-A and SSC-A. An extra level of gating based on FSC-H allowed us to identify and bias towards the collection of singletons. We attempted to sort single cells into wells of a 96-well plate. Approximately 15% of the wells appeared to get single cells, but subsequent genotyping revealed that the other wells got multiple cells. We wanted the cells intact for phenotyping and thus did not remove the flagella prior to FACS sorting, which may have contributed to the problem.

### Microscopic examination of sperm

We ran two experiments. In each, sperm was collected in 1× Hank’s and diluted with equal volumes of water, or water with 80 μM Hoechst 33342, bringing the concentrations of Hank’s to 0.5× and the dye to 40 μM. We transferred 1 μl of the suspension into eight different wells of a 12-well multitest slide (MP Biomedicals) and covered with a coverslip. Wells on the multitest slide are 17 μm deep and the 1 μl volume nearly covered the whole area of the well. Wells were scanned once each, moving the stage from right to left. At each position, the field was photographed (Firefly USB2, FLIR Vision). Every cell in the fields of vision was counted from the photographs. Clusters of cells were counted and their contents enumerated if they contained six or fewer cells. The contents of rare larger clusters were difficult to evaluate and were not counted.

### Amplification and qPCR genotyping

The FACS-sorted cells were lysed for 10 min at 65 °C in 10 μl of 1× Flexi buffer (Promega) containing 8 μg of proteinase k (Qiagen); the proteinase k was subsequently deactivated at 94 °C for 5 min. The well contents were then treated to a whole genome primer extension preamplification (PEP [15, 16]) to obtain product for qPCR. We used a Roche LightCycler480 for qPCR and primers and probes synthesized by IDT (see Additional file 3 for sequences). Samples were genotyped separately for the two loci.

### qPCR analysis

To develop a method for estimating relative concentrations of cave and surface alleles in the wells, we performed a titration analysis of *oca2* by qPCR, using mixtures of genomic DNA purified from fin clips from the parents of the hybrids. We ran reactions with pure surface or pure cave DNA and seven other samples with stepwise titration of the two (Additional file 6). Each of the nine samples was run at four concentrations (16, 8, 4, and 2 ng template DNA per reaction), and in duplicate. A scatter plot of the end-point genotyping results exhibited a fan-shaped array (Additional file 7) which suggested a simple trigonometric procedure for representing the relative concentrations of the two alleles.

The relative concentrations of cave and surface alleles in each well were transformed in Excel into equivalent angles from the origin based on their end-point coordinates *X* and *Y*, as =ATAN(*Y*/*X*)*57.2958. Steeper angles were associated with higher proportions of the cave allele, less steep angles with higher proportions of the surface allele.

The analysis was based on the angularly transformed data and was designed to screen out wells that had received many sperm cells and were thus uninformative. To do this, we limited inclusion in the analysis to wells in which both the *oca2* and *mc1r* angles were extremes. The stringency level for inclusion was varied in 2.5° steps from 15° to 30°. For example, when stringency was set at 15°, all data points between 0° and 15°, as well as all those between 75° and 90°, were admitted to consideration. For inclusion in the analysis, both loci had to pass the screen. Included wells were designated as HOM or HET based on concordance of c > s or s > c at the two loci. The significance of any deviation from a 1:1 ratio was tested by the binomial. When stringency was set at 15°, approximately 98% of the data were screened out. When stringency was relaxed to 30°, approximately 86% of the data were screened out. All reported significance values are two-tailed.

The study was not blind, as the analyst knew which populations were being analyzed. Because effect sizes were unknown, a fully informed power analysis could not be performed to guide choice of sample size. However, previous work ([12], Table S3) had estimated that the F_2_ of a Pachón cave × surface hybrid cross had a genome wide overall HOM:HET ratio of 53:47. Using the difference (6%) as a rough estimate of effect size, a power of 80% to detect a difference with 95% confidence would require a sample size of 1087 (https://onlinelibrary.wiley.com/doi/10.1002/9781118445112.stat07091).

### Simulation

The experiment was modeled in Excel. Five or 10 haplotypes were determined using the rand() function which returns a number between 0 and 1 with a uniform distribution. The haplotypes were determined in both unbiased and biased replicates. For the unbiased model, all four haplotypes had probabilities set at 0.25. For the first biased model, the two HET combinations had probabilities of 0.3 and the two HOM combinations had probabilities of 0.2. For the second biased model, the probabilities were 0.275 and 0.225 respectively.

The different models were all run 1000 times, a number chosen to be close to the sample sizes in the actual experiments (960, 1152, and 767). Each result is analogous to a single well in the actual experiment. For each “well” in the simulation, its contents were represented as the number of cave alleles for *mc1r* and the number of cave alleles for *oca2*. Thus, while the wells were originally constituted with 5 or 10 haplotypes, the haplotypic information was discarded and only the allelic proportions were retained for further analysis. This essentially matched the situation in the actual experiments.

For the analyses, the 1000 replicates were screened to remove intermediates. We used two filters: for the more stringent one, any well with a cave allelic frequency greater than 0.2 or less than 0.8, at either locus, was discarded. For the relaxed stringency, the filters were set to 0.3 and 0.7, respectively. (The relaxed stringency could only be imposed on the 10-cell model because the 5-cell model does not have values of 0.3 and 0.7.)

This screening is analogous to the trigonometric screens in the analysis of the real experiments. It is noteworthy that the percentage of wells that passed the screens in the simulation (ranging from 1.0 to 16.5) was almost identical to the range of percentages of wells that passed the screens in the experiments (1.3 to 14.5). This suggests that the values chosen for the modeling were plausible.

The “wells” that passed the screening were then designated HOM if the proportions of cave alleles at the two loci were either both high or both low. They were designated HET if one was high and the other was low. The results were tested for inequalities of HET and HOM contents using the binomial test. Analyses of simulations with biased inputs recovered biased outputs. Simulations in which the input was HET = HOM had outputs that were close to 1:1 and were statistically insignificant. Thus, the analysis does not manufacture spurious significance. The Excel files used for the simulations are in Additional files 4 and 5.

## Additional files

Additional file 1: Evidence from dye fluorescence that the three subpopulations correspond primarily to single cells, and clusters of two and three cells. (DOCX 376 kb)

Additional file 2: Excel workbook which enumerates the phenotypes and numbers of sperm, as single cells or clusters of cells in untreated and dye-treated samples. (XLSX 11 kb)

Additional file 3: Sequences of primers and probes for *mc1r* and *oca2* cave and surface alleles. (DOCX 26 kb)

Additional file 4: Excel workbooks that were used for the simulation experiments. Each file has an overview sheet that details the analysis structure. (XLSM 589 kb)

Additional file 5: Excel workbooks that were used for the simulation experiments. Each file has an overview sheet that details the analysis structure. (XLSM 291 kb)

Additional file 6: Titration stages for qPCR of *oca2* alleles. (DOCX 26 kb)

Additional file 7: qPCR results for the titration of cave against surface alleles of *oca2* according to the titration plan in Additional file 6. (JPG 115 kb)

Additional file 8: Original data. Each subfolder has two ixo files output by the Roche LightCycler480. The two files cover qPCR results for *oca2* and *mc1r*. Subfolder names end in 1, 2, or 3, denoting which subpopulation of sperm was analyzed. (ZIP 10643 kb)

Additional file 9: Data extracted from the ixo files; the data are in Excel files with the endpoint analyses for all the individuals in all the three subpopulations. (ZIP 252 kb)

Additional file 10: Data extracted from the ixo files; the data are in Excel files with the endpoint analyses for all the individuals in all the three subpopulations. (ZIP 210 kb)

Additional file 11: Data extracted from the ixo files; the data are in Excel files with the endpoint analyses for all the individuals in all the three subpopulations. (ZIP 128 kb)

Additional file 12: Excel file with 10 worksheets documenting the statistical analyses of the relative allelic contents of the wells for each subpopulation. Worksheets 2, 5, and 8 contain the data on allelic proportions for the three subpopulations. Worksheets 3, 6, and 9 convert the relative allelic concentrations of the two loci into designations of HOM or HET for each well. These worksheets are interactive; change the integer in the yellow box and read out tallies of HOM and HET in the green boxes (maximum integer value = 30). Worksheets 4, 7, and 10 contain the results of the analyses for the three subpopulations. Worksheet 11 compares results for the three subpopulations and also for subpopulations 2 and 3, combined. (XLSX 801 kb)
