# Author Correction: Unique sperm haplotypes are associated with phenotypically different sperm subpopulations in *Astyanax* fish

**DOI:** 10.1186/s12915-023-01631-0

**Published:** 2023-05-29

**Authors:** Richard Borowsky, Alissa Luk, Xinjian He, Rebecca S. Kim

**Affiliations:** Department of Biology, New York University, New York, USA

**Correction: BMC Biol 16, 72 (2018)**

**https://doi.org/10.1186/s12915-018-0538-z**

The original article [1] contains a minor typo in Table 2.

The haplotype motif, M_S_M_O_ is erroneously displayed several times in both the fourth column of the table and the final line of the table caption.

The motif should instead read as M_S_O_S_.
