# Supplementary material for: Unique sperm haplotypes are associated with phenotypically different sperm subpopulations in Astyanax fish
Source: BMC Biol. 2018 Jul 5;16:72. doi: 10.1186/s12915-018-0538-z (PMC6032774; doi:10.1186/s12915-018-0538-z)

Additional File 1:

Figure 1 in the main body of the paper shows that in the absence of Hoechst 33342 dye treatment the plot of SSC-A against FSC-A reveals one population. Treated with dye, however, multiple sub populations are revealed. This figure is embedded here for quick reference.


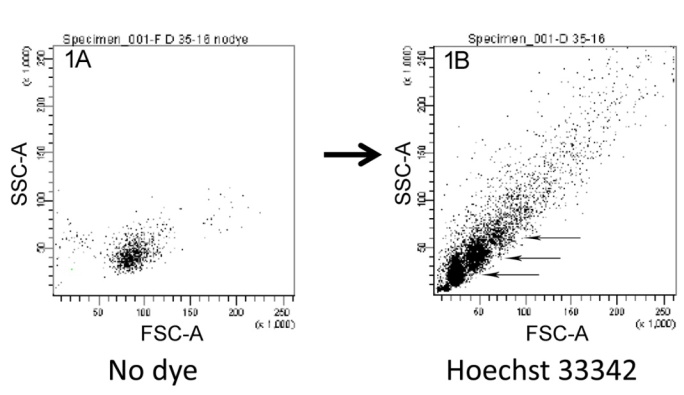


Figure 1 caption: 1A) Sperm from a hybrid male from one population in 0.4X Hanks balanced saline. 1B) Sperm from the same male treated with Hoechst 33342 dye (40μM).

The basis for the manifestation of new sub populations is clumping. The two plots in blue below show a histogram of Hoechst 33342 fluorescence and a plot of fluorescence against FSC-A. Both plots reveal three main populations (and a fourth minor peak). The mean fluorescence of peak 2 is twice that of peak 1, while the third and fourth peaks are 3 and 4 times the fluorescence of peak 1, respectively. Because the amount of fluorescence is proportional to the quantity of DNA, populations 1, 2, and 3, correspond to single cells, doubletons and triplets (and higher).


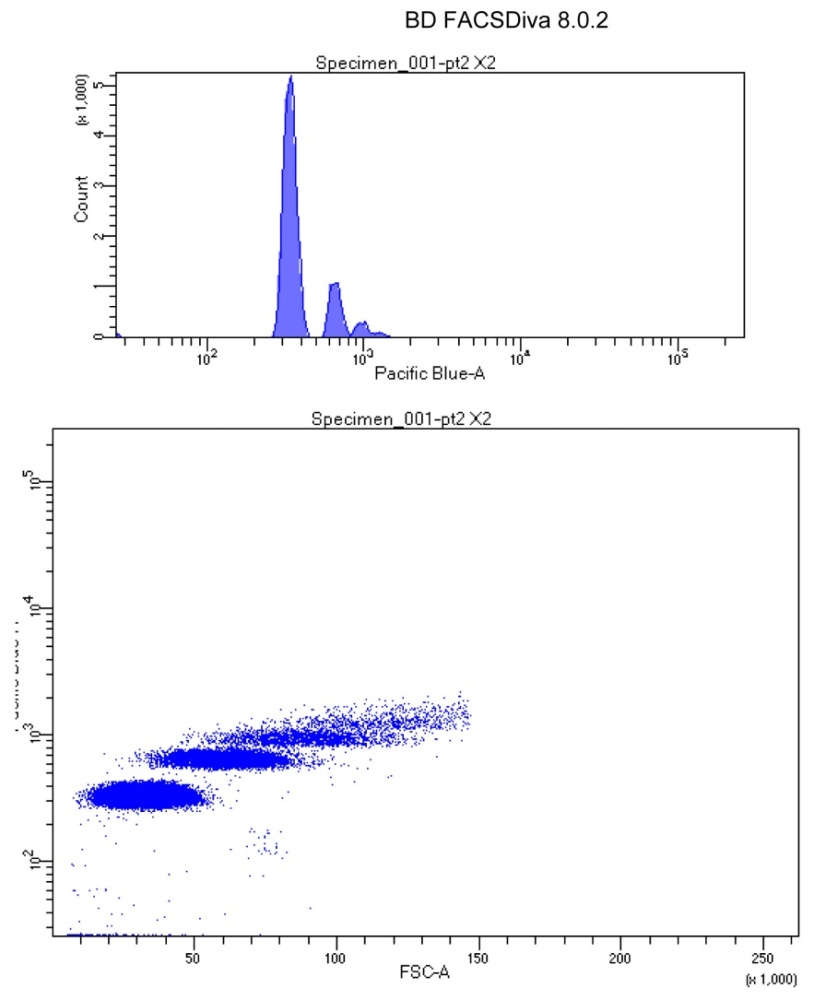


The upper left panel on the figure below shows a plot of SSC-A against FSC-A in the visible range, which is what we worked with for FACS sorting. It exhibits a clear sub population near the origin, a second one above 50 on the abscissa, and a scattering of cells mainly over 80 on both axes. These correspond to what we call subpopulations 1-3. The large middle panel in the figure below shows the color coded gating of cells based on fluorescence, where the three gates mark single cells, doubletons, and triplets (plus higher). The top right panel is the same as the one to its left, except it is painted with the colors from the gating. There is an almost perfect correspondence between the populations based on forward and side scatter and the cells’ clusterings.


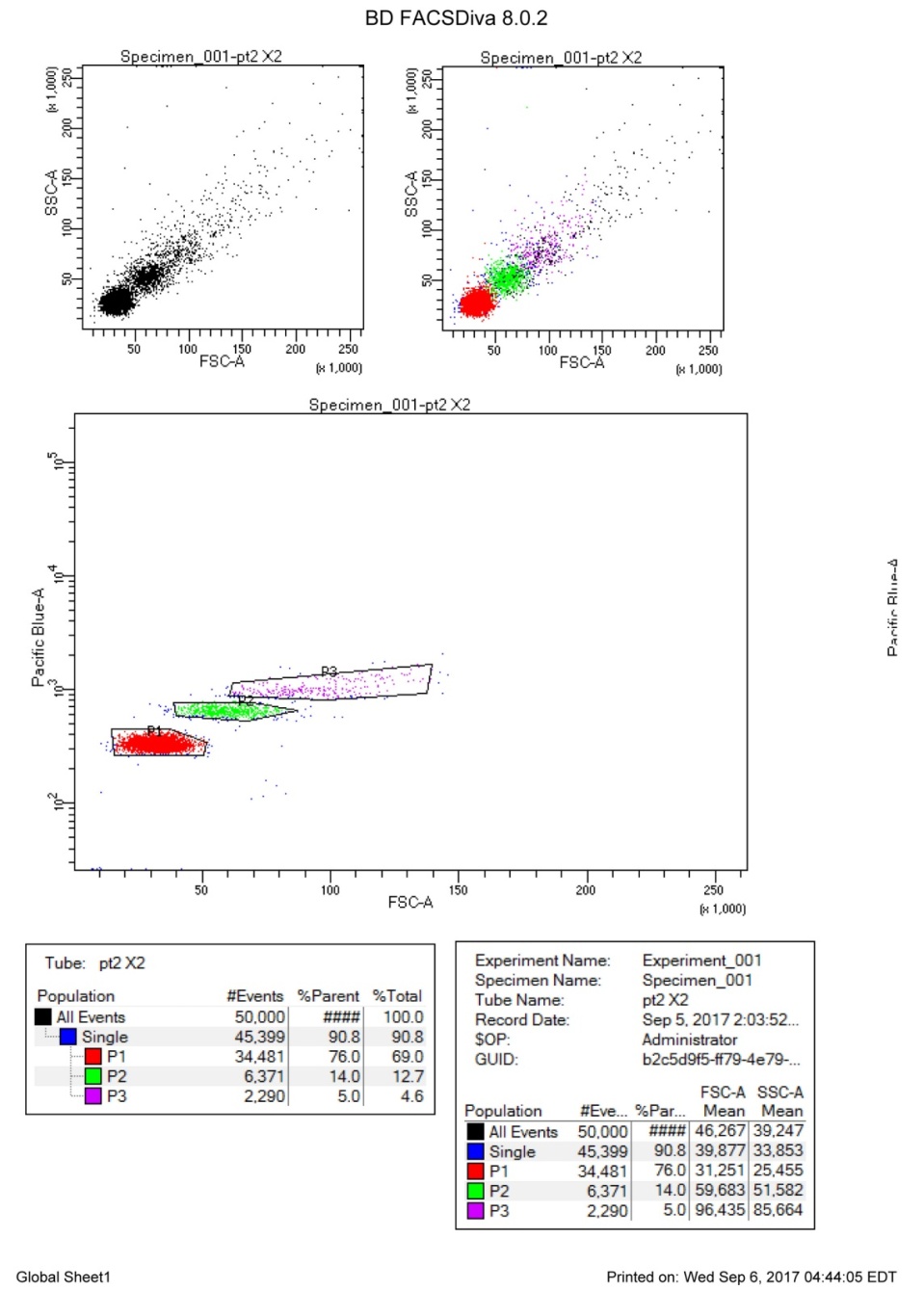

Supplement: Supplementary file 1 — Evidence from dye fluorescence that the three subpopulations correspond primarily to single cells, and clusters of two and three cells. (DOCX 376 kb) [file 12915_2018_538_MOESM1_ESM.docx]
