# Supplementary material for: Unique sperm haplotypes are associated with phenotypically different sperm subpopulations in Astyanax fish
Source: BMC Biol. 2018 Jul 5;16:72. doi: 10.1186/s12915-018-0538-z (PMC6032774; doi:10.1186/s12915-018-0538-z)
Supplement: Supplementary file 3 — Sequences of primers and probes for mc1r and oca2 cave and surface alleles. (DOCX 26 kb) [file 12915_2018_538_MOESM3_ESM.docx]

Additional File 3: Primer and probe sequences for *oca2* and *mc1r* allelic genotyping by qPCR in *Astyanax mexicanus*.

Oca2 Surface Set

Primer1 GACCCTCTCTGACACCAATTATG

Primer2 TGACAGTTCCATTCCTGTTCTG

Probe /5’HEX/TGGCTCCGG/ZEN/CAGAATCAGAGAAAT/3IABkFQ/

Oca2 Cave Set

Primer1 AAGATAGAGGACCCTCTCTGAC

Primer2 CCACTTGACAGTTCCATTCCT

Probe /5’6-FAM/ACCAACACC/ZEN/GGCAGAATCAGAGAA/3IABkFQ/

Mc1r Surface Set

Primer1 GGAACTCTGAGTCACCATGAAC

Primer2 TGCCCGTGGCATTCATATC

Probe /5’TET/AACACCCTG/ZEN/CACCACCACTTGG/3IABkFQ/

Mc1r Cave Set

Primer1 GGAACTCTGAGTCACCATGAAC

Primer2 TGCCCGTGGCATTCATATC

Probe /5’Cy5/AACACCCTGCACCACTTTGGC/3IAbRQSp/

Additional File 3 Caption: The excitation and detection wavelengths for the four alleles were: HEX 535 and 553nm; FAM 494 and 518nm; TET 498 and 580nm; CY5 618 and 660nm. Primers and probes synthesized by IDTDNA.
