# Supplementary material for: Unique sperm haplotypes are associated with phenotypically different sperm subpopulations in Astyanax fish
Source: BMC Biol. 2018 Jul 5;16:72. doi: 10.1186/s12915-018-0538-z (PMC6032774; doi:10.1186/s12915-018-0538-z)
Supplement: Supplementary file 6 — Titration stages for qPCR of oca2 alleles. (DOCX 26 kb) [file 12915_2018_538_MOESM6_ESM.docx]

Additional file 6: Titration plan for mixing cave and surface alleles at *oca2* for qPCR endpoint analysis.

C/S Mix P(C) P(S)

1 0. 985 0. 015

2 0. 941 0. 059

3 0. 800 0. 200

4 0. 500 0. 500

5 0. 200 0. 800

6 0. 059 0. 941

7 0. 015 0. 985

Additional file 6 caption: Cave/Surface (C/S) mixes 1 to 7 are labeled in Additional file 7. P(C) and P(S) give the proportions of cave and surface alleles in mixtures of genomic DNA prepared from homozygous cave and surface fish.
