# Supplementary figures and images for: Unique sperm haplotypes are associated with phenotypically different sperm subpopulations in Astyanax fish
Source: BMC Biol. 2018 Jul 5;16:72. doi: 10.1186/s12915-018-0538-z (PMC6032774; doi:10.1186/s12915-018-0538-z)

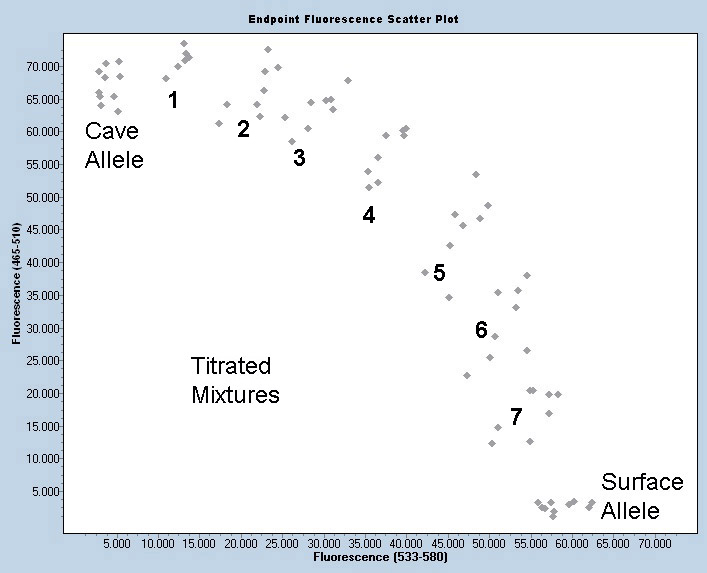

Supplement: Supplementary file 7 — qPCR results for the titration of cave against surface alleles of oca2 according to the titration plan in Additional file 6. (JPG 115 kb) [file 12915_2018_538_MOESM7_ESM.jpg]
